# Distance correlation application to gene co-expression network analysis

**DOI:** 10.1186/s12859-022-04609-x

**Published:** 2022-02-21

**Authors:** Jie Hou, Xiufen Ye, Weixing Feng, Qiaosheng Zhang, Yatong Han, Yusong Liu, Yu Li, Yufen Wei

**Affiliations:** 1 College of Intelligent Systems Science and Engineering, Harbin Engineering University, Nantong Street, Harbin, China; 2 College of Science, Heilongjiang Bayi Agricultural University, Xinfeng Road, Daqing, China; 3 School of Computer Engineering, Jiangsu Ocean University, Cangwu Road, Lianyungang, China; 4 College of Science, Northeast Forestry University, Hexing Road, Harbin, China

**Keywords:** Gene expression, Distance correlation, WGCNA, Enrichment analysis

## Abstract

**Background:**

To construct gene co-expression networks, it is necessary to evaluate the correlation between different gene expression profiles. However, commonly used correlation metrics, including both linear (such as Pearson’s correlation) and monotonic (such as Spearman’s correlation) dependence metrics, are not enough to observe the nature of real biological systems. Hence, introducing a more informative correlation metric when constructing gene co-expression networks is still an interesting topic.

**Results:**

In this paper, we test distance correlation, a correlation metric integrating both linear and non-linear dependence, with other three typical metrics (Pearson’s correlation, Spearman’s correlation, and maximal information coefficient) on four different arrays (macrophage and liver) and RNA-seq (cervical cancer and pancreatic cancer) datasets. Among all the metrics, distance correlation is distribution free and can provide better performance on complex relationships and anti-outlier. Furthermore, distance correlation is applied to Weighted Gene Co-expression Network Analysis (WGCNA) for constructing a gene co-expression network analysis method which we named Distance Correlation-based Weighted Gene Co-expression Network Analysis (DC-WGCNA). Compared with traditional WGCNA, DC-WGCNA can enhance the result of enrichment analysis and improve the module stability.

**Conclusions:**

Distance correlation is better at revealing complex biological relationships between gene profiles compared with other correlation metrics, which contribute to more meaningful modules when analyzing gene co-expression networks. However, due to the high time complexity of distance correlation, the implementation requires more computer memory.

**Supplementary Information:**

The online version contains supplementary material available at 10.1186/s12859-022-04609-x.

## Background

The general insight in systems biology is that genes and proteins act together in intricate networks, rather than in isolation [1]. Networks are widespread in biomedicine, such as the gene co-expression network, regulatory network metabolism network and protein-protein interaction network [2]. A key step in the analysis of large genome-wide gene expression datasets is the grouping of genes into co-expression modules using module detection methods [3]. Genes are represented by nodes, and connecting the node genes which are significantly co-expressed across appropriately chosen samples [4].

To construct and analyse a gene co-expression network, it is necessary to assess the interactions between two genes. Such interactions are measured by calculating the correlation coefficients of different gene expression profiles. Detecting and evaluating dependencies between variables usually requires the definition of ‘ distance’ or ‘similarity’ [5]. In the gene co-expression network, the overall shapes of gene expression patterns (or profiles) are of greater interest than the individual magnitudes of each feature [6]. The gene modules obtained by gene co-expression network analysis should represent highly correlated genes which are also called co-expressed genes [7]. Therefore, we should apply a metric between the gene based on ’similarity’ measure rather than using ’distance’ depending on the gene co-expression network. There are many methods to calculate the correlation coefficients between genes, with the most popular being Pearson correlation and Spearman’s rank correlation. Pearson correlation measures the strength of the linear relationship between two random variables, whereas Spearman’s rank correlation evaluates how well the correlation between two variables can be formulated by a monotonic function [8, 9]. However, linear and monotonic dependence are not the only ones observed in a real biological system, there are many other complex relationships observed in biological systems. Therefore, if the method of correlation measure is limited to linear dependence measures in the construction of gene co-expression networks, the ability of gene co-expression networks to recreate the accurate network and identify the appropriate gene modules will also be limited. To overcome this barrier, additional appropriate methods are needed to measure the complex relationships between genes.

The Pearson correlation coefficient is the most common default measure among gene co-expression network analysis methods [10, 11]. Several assumptions made with respect to Pearson correlation limit its effectiveness. (i) The Pearson correlation coefficient is usually not suggested for non-normally distributed data [12]. (ii) Pearson correlation, which only captures linear relationships between two given components, measures the degree of a linear relationship between two real-valued variables [13]. That is, the linear relationships between two variables should be known, after which Pearson correlation can be used to assess how well the relationship between the two variables can be described by a linear equation. However, the relationships between genes are complex, and only partly explained by linear relationships. (iii) The Pearson coefficient is sensitive to outliers. For example, if one sample has a very high expression for two genes, the Pearson correlation coefficient can approach +1 due to just one outlier, falsely indicating a strong correlation, when in fact it does not exist [14]. (iv) A zero value for the Pearson coefficient reveals there isn’t a linear correlation between the variables, however, it does not show that the variables are independent. A classic example is to define $Y=X^2$ where *X* is a random variable on $[-1, 1]$. *X* and *Y* are not independent, but the value for the Pearson correlation coefficient between *X* and *Y* is zero.

A robust alternative to the Pearson correlation coefficient is the Spearman correlation coefficient which can be applied to non-normally distributed data since it is more robust to outliers. As the Spearman correlation coefficient has less statistical power than the Pearson coefficient, the Pearson coefficient is recommended if the data are normally distributed [15]. Moreover, since gene data are continuous, Spearman correlation must convert continuous into rank data, leading to a loss of the original information. For continuous data, Spearman correlation has lower accuracy than Pearson correlation.

There are many other metrics in addition to the Pearson correlation coefficient and Spearman correlation coefficient, such as the maximal information coefficient (MIC) [16] and distance correlation [17]. Maximal information component analysis (MICA), which combines MIC with an Interaction Component Model, has been proposed in [18]. This algorithm can obtain improved modules when the networks contain confounding factors. However, it has been indicated that there are no mathematical arguments for MIC, and its results are based solely on the analysis of simulated data in the literature [19–21]. In practice, MIC may provide a high score for two variables even if they are not correlated, thus creating a false correlation between two variables.

Distance correlation is a statistical measure of the correlation proposed by Szkely et al. [17]. In contrast to Pearson correlation, which can only detect linear relationships, distance correlation measures the degree of all types of possible relationships between two genes. Generally speaking, compared with classical Pearson correlation, distance correlation has some distinct advantages: it does not assume normality, it can measure a nonlinear relationship between two variables, the presence of outliers has a reduced influence on distance correlation, and the distance correlation is zero only if the random vectors are independent. Distance correlation is a valuable, practical, and natural tool in data analysis [17]. Inspired by MICA, we incorporated distance correlation into WGCNA to construct a distance correlation-based WGCNA (DC-WGCNA) algorithm for gene co-expression analysis. In DC-WGCNA, the correlation coefficients between the gene expression profiling data are calculated by distance correlation, and the other process of DC-WGCNA is identical to the traditional WGCNA except for the different correlation coefficients. To illustrate the performance of distance correlation, the three most popular used and representative correlation coefficients were selected for comparison: Pearson product-moment correlation, Spearman’s rank-order correlation, and maximal information coefficient (MIC) based on information entropy.

In this study, we mainly used four datasets, macrophage [22–24] and liver [25, 26] datasets were from microarray datasets. The data were normalized by the robust multi-array average (RMA) method. Here, we used the macrophage dataset comprising 329 samples, for which 3611 genes were retained. Simultaneously, the liver dataset comprised 288 samples and 3089 genes. Cervical cancer and pancreatic cancer datasets were RNA-seq datasets derived from The Cancer Genome Atlas (TCGA). These datasets are transformed logarithmic and RSEM normalized. We selected 3808 genes in 308 samples for the cervical cancer dataset. For the pancreatic cancer dataset, there are 3029 genes in 183 samples.

## Results

**Fig. 1:**
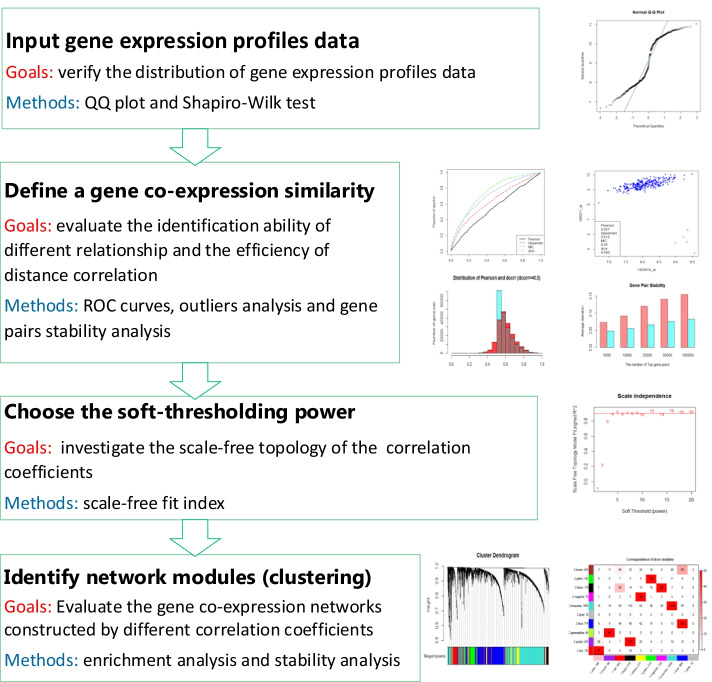
Flowchart of the study. This flowchart presents a brief overview of the evaluation goals and methods for distance correlation in each main steps of WGCNA

In this section, we first illustrate that distance correlation is suitable for measuring the correlation between two genes. Simultaneously, distance correlation is compared with the other three typical correlation analysis methods named Pearson correlation, Spearman correlation, and MIC. Then, we construct a distance correlation-based WGCNA (DC-WGCNA) algorithm for gene co-expression analysis by replacing the Pearson correlation in WGCNA with distance correlation. Finally, we validate the performance of the new algorithm based on scale-free topology (SFT) fit, clustering results, enrichment analysis, and module stability by analysing gene expression profiles using four datasets from microarray data and RNA-seq data. In Fig. 1, we provide a flowchart of this study.

### Distance correlation is distribution free

**Fig. 2:**
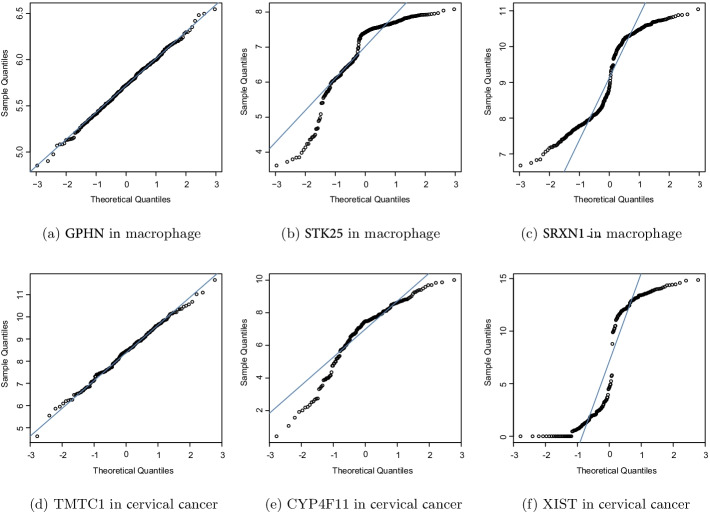
Normal QQ plot of gene expression profiles obtained from the macrophage and cervical cancer datasets. The gene distribution is not only normal but also includes other types of probability distribution

A normal distribution is not a requirement for using the Pearson correlation coefficient, but the testing for statistical significance of the correlation may be reduced, so the Pearson correlation coefficient is usually not suggested for non-normally distributed data [12, 15, 27, 28]. The gene data we used were usually log-transformed to achieve a normal distribution. However, most genes are in fact not normally distributed even after log transformation. To verify that the data were normally distributed, we show the normal quantile-quantile (QQ) plots of the gene expression profile obtained from the macrophage and cervical cancer datasets in Fig. 2. Each sub-figure of Fig. 2 is a normal QQ plot of one gene expression profile. A normal QQ plot comparing a gene expression profile on the vertical axis to a standard normal population on the horizontal axis is shown. In the QQ plots, normally distributed data appear as an approximately straight line. The GPHN gene shown in Fig. 2a is approximately normally distributed. However, in Fig. 2b, c, the points are not clustered on the $45^\circ$ line, but rather follow a curve, suggesting that the genes are not normally distributed. The data for these two microarrays show skewness and a bimodal distribution, respectively. These distributions were also observed in the RNA-seq data for cervical cancer, where the results shown in Fig. 2d are approximately normally distributed, in Fig. 2e show a skewness distribution, and in Fig. 2f show a bimodal distribution.

Using the Shapiro-Wilk test [29], we calculated the percentage of genes with a p-value greater than 0.01, which were considered to be normally distributed. In the macrophage dataset, approximately 34.26% of genes were normally distributed, while this value was 30.88% in the liver dataset, 37.65% in the cervical cancer dataset, and 23.21% in the pancreatic cancer dataset. Thus, most of the genes in these four datasets were not normally distributed. Since distance correlation does not require any distributional assumption [17], testing for the statistical significance of distance correlation will not be less even if the data are non-normally distributed, which differs from the Pearson coefficient. Thus, a reasonable measurement value is given by the distance correlation, making distance correlation more suitable for measuring the dependence between two genes.

### Distance correlation better fits complex relationships

**Fig. 3:**
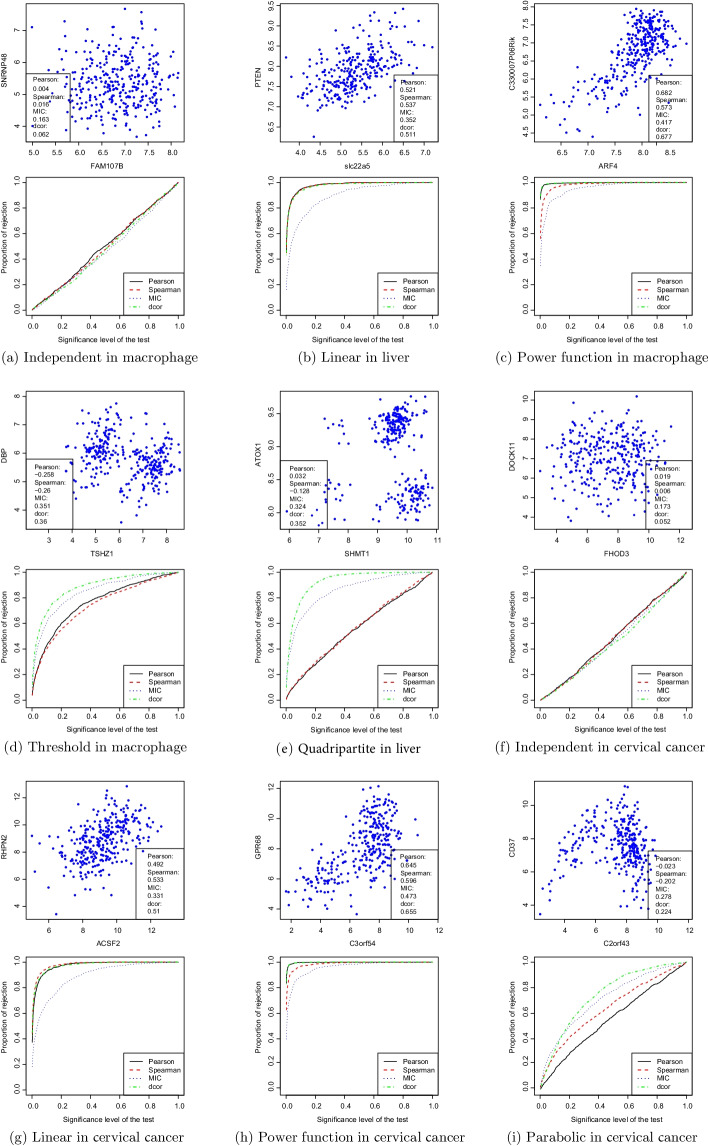
Complex relationships observed between pairs of genes and corresponding ROC curves (sample size n = 30). The distance correlation can well-represent complex relationships between genes

Complex relationships between genes are pervasively observed in biological systems. In Fig. 3, we provide several examples of relationships observed between pairs of genes from microarray data and RNA-seq data. These relationships of gene pairs were selected based on the Pearson correlation values, the distance correlation values, and the difference between them. Specifically, a small distance correlation value indicates an almost independent relationship. A small difference between the Pearson and distance correlation values might associate with linearity. A greater Pearson coefficient than distance correlation value indicates the potential for outliers in the direction of the regression line of the normal data. A smaller Pearson coefficient than the distance correlation value indicates a nonlinear relationship or outliers in the normal direction of the regression line.

s shown in Fig. 3a, the relationship between the two genes was approximately independent. Figure 3b shows a linear and monotonic relationship between the two genes. In Fig. 3c, a significant power function relationship can be observed between the two genes, suggestive of a non-linear and monotonic relationship. Figure 3d, e show the threshold and quadripartite relations, respectively, which are nonlinear and non-monotonic. For RNA-seq data cervical cancer, independent, linear and power function relationships can also be observed in Fig. 3f–h. Figure 3i shows the parabolic relation, which is nonlinear and non-monotonic. To evaluate the identification ability of different correlation coefficients, we correspondingly show the receiver operating characteristic (ROC) curves of the four methods (sample size of 30) in Fig. 3. Table 1 provides the areas under the ROC curves obtained by applying each method for every gene relationship corresponding to Fig. 3. For each varied sample size *n* ($n=15, 30, 60$), we applied the statistical tests (null hypothesis: two variables independent, alternative hypothesis: dependent). The ROC curves were constructed by repeating the above procedure 1000 times. In the ROC curves, the x-axis denotes the significance level (P-value), and the y-axis denotes the proportion of the rejected null hypothesis. The ROC curves show the proportion of the rejected null hypothesis on different significance levels. When the two variables are dependent, the higher the proportion of rejected null hypothesis, the stronger is the correlation coefficient identification ability at the same significance level. Then, if the power curve of the measure increases rapidly, the responding area close to 1 denotes high power. A ROC curve close to diagonal $45^\circ$ or an area under the ROC curve close to 0.50 is equivalent to random decisions [28].

**Table 1:** Areas under the ROC curves for different relationships obtained by each method

Type of association	Sampling number	Pearson	Spearman	MIC	Dcorr
Independent in macrophage	15	0.497640	0.490286	0.452818	0.473846
	30	0.497310	0.490880	0.466964	0.476830
	60	0.471582	0.455186	0.441598	0.427666
Linear in liver	15	0.885432	0.870752	0.777636	0.876410
	30	0.978370	0.977436	0.894208	0.974430
	60	0.999530	0.999334	0.967184	0.999214
Power function in macrophage	15	0.951840	0.879982	0.812880	0.958504
	30	0.996426	0.983466	0.953426	0.997150
	60	1.000000	0.999616	0.995276	0.999992
Threshold in macrophage	15	0.639518	0.614368	0.642340	0.730636
	30	0.739068	0.724708	0.833202	0.871750
	60	0.890084	0.872332	0.946704	0.976500
Complex in liver	15	0.580804	0.546426	0.636092	0.816852
	30	0.559342	0.567496	0.860154	0.930690
	60	0.533458	0.626920	0.955248	0.991334
Independent in cervical cancer	15	0.488086	0.486846	0.447234	0.476932
	30	0.486354	0.484166	0.468006	0.457722
	60	0.467092	0.458260	0.408864	0.389114
Linear in cervical cancer	15	0.867518	0.864164	0.777916	0.861502
	30	0.974396	0.980996	0.889826	0.975538
	60	0.999226	0.999606	0.964436	0.999472
Power function in cervical cancer	15	0.957340	0.903698	0.840232	0.952058
	30	0.997856	0.985916	0.959978	0.997838
	60	1.000000	0.999926	0.996460	0.999998
Parabolic in cervical cancer	15	0.564414	0.574200	0.557816	0.641100
	30	0.556910	0.643414	0.714194	0.744158
	60	0.531748	0.756100	0.831724	0.877858

Next, we will discuss the ability of the four correlation methods to identify the relationship of various variables by using the ROC curves in Fig. 3 and the areas under the ROC curves in Table 1. The results shown in Fig. 3a is the independent relationship in the macrophage: the ROC curve of each algorithm is close to the diagonal $45^\circ$ and the area under the curve is close to 0.5. All four methods failed to identify any association, which correctly reflected the independent relationship between the two genes. The results are shown in Fig. 3b is the linearity in the liver: the Pearson, Spearman, and distance correlation could quickly identify a linear correlation, but the MIC was relatively weaker in terms of identifying a linear relationship. Figure 3c shows the power function relationship in the macrophage: Pearson and distance correlation were the most powerful methods to identify this relationship, while Spearman was the second most powerful and MIC the weakest. It should be noted that the Pearson coefficient, which is generally considered to have a strong ability to recognize linear relationships, in this case, Fig. 3c can identify the non-linear monotonic relationships with high power. Figure 3d, e respectively show thresholds relationship in macrophage and quadripartite relationship in the liver, both of which were non-linear and non-monotonic. For these kinds of relationships, distance correlation has the strongest identification ability, followed by MIC and Pearson and Spearman as the weakest (lowest power). For RNA-seq cervical cancer data, independent, linear and power function relationships are also observed in Fig. 3f–h. The ability to identify the relationship is consistent with the microarray data. Figure 3i reflects the parabolic relationship, which is non-linear and non-monotonic. For this kind of relationship, distance correlation has the strongest identification ability, followed by MIC, Spearman, and Pearson as the weakest (lowest power).

### Distance correlation is robust to outliers

**Fig. 4:**
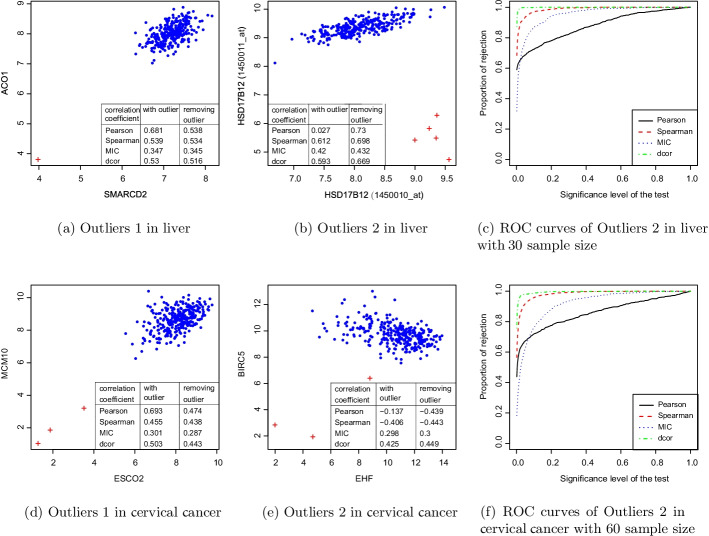
Distance correlation is robust to outliers. The Pearson correlation coefficient is too sensitive while other correlation coefficients are robust to outliers

In statistics, an outlier is a data point that differs significantly from other observations [30]. We selected four pairs of genes from the liver and cervical cancer dataset to observe the effect of outlier points on correlation coefficients. In Fig. 4a, b, d, e, the values of four correlation coefficients with and without outliers are shown, respectively. In Fig. 4a, a point in the bottom left corner was determined as the outlier using the local outlier factor (LOF) algorithm [31]. The LOF algorithm is implemented using the ‘lofactor’ Function in the R package ‘DMwR’ [32]. All the correlation values decreased when the outlier points were removed, and the correlation coefficients became larger when the outliers were in the direction of the regression line of the normal data. In Fig. 4b, five points in the bottom right corner were determined as outliers using the LOF algorithm. Moreover, these outliers were in the normal direction of the regression line and maintained a good distance from it. The correlation coefficients with outliers were smaller than those without outliers. By comparing the two pairs of data, the Pearson coefficient changed the most, while the other three correlation coefficients did not vary as sharply. These results indicate that Pearson correlation is too sensitive to outliers even if there are few, but distance correlation and other correlation coefficients are not as sensitive. Researchers may incorrectly identify the correlation of two genes if the analysis is performed only according to Pearson coefficient values in the presence of outliers. From the ROC curve shown in Fig. 4c corresponding to Fig. 4b, the correlation identification ability of distance correlation is strongest, while the Pearson coefficient is weakest in the presence of gene correlations and outliers.

The phenomenon in which outliers lie in the direction of the regression line and in the normal direction can also be found in the RNA-seq data for cervical cancer (see Fig. 4d, e). This conclusion was consistent with the microarray data. Pearson correlation is too sensitive to outliers, and the correlation identification ability of distance correlation is strongest.

For most cases shown in Table 1, if two genes were dependent, the probability of type I error decreased as the sample size increased, the probability of rejecting $H_0$ increased, and the area under the curve increased. As a result, relationships between genes become easier to recognize when the samples size is increased. However, in the case of outliers in Table 2, the area under the curve obtained by the Pearson coefficient decreased with increasing sample size. Thus, the ability of the Pearson coefficient to identify the relationship between genes decreased because the probability of sampling outliers will be higher with increasing sample size. The Pearson coefficient was too sensitive to outliers; thus, its ability to identify the correlation between two genes was weakened. For the four datasets used in this paper, when outliers existed, the distance correlation was robust to the outliers, and the ability to identify correlations between genes was strongest.

**Table 2:** Areas under ROC curves in the presence of outliers using each method

Type of association	Sampling number	Pearson	Spearman	MIC	Dcorr
Outliers 2 in liver	15	0.948112	0.908492	0.850688	0.974058
	30	0.879612	0.988404	0.954280	0.999168
	60	0.707942	0.999900	0.993774	1.000000
Outliers 2 in cervical cancer	15	0.812824	0.740076	0.677720	0.814222
	30	0.912892	0.891988	0.804872	0.948394
	60	0.859602	0.984052	0.920278	0.995328

Based on the above findings, distance correlation seemed more appropriate than other correlation coefficients for the identification of relationships between genes. In this paper, distance correlation was applied to construct the gene co-expression network, so we will discuss the difference between distance correlation and the classical Pearson coefficient, which is used in WGCNA. Additionally, the difference between DC-WGCNA and WGCNA is also compared and analysed.

### Effects of distance correlation on different datasets

**Fig. 5:**
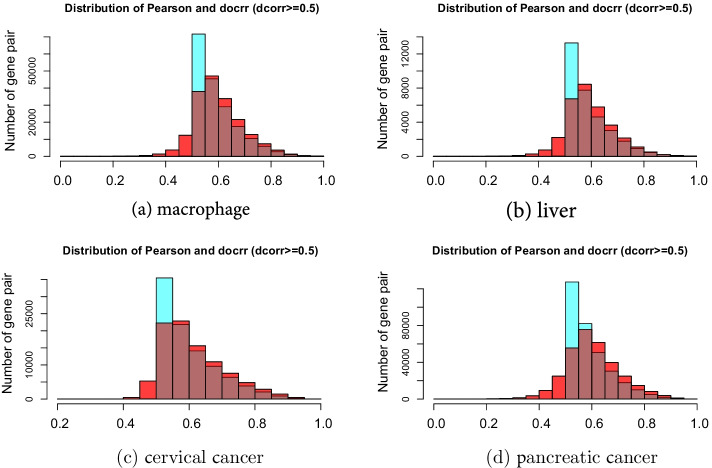
Histograms of correlation coefficients for interactions with high distance correlation scores ($>0.5$). The red border in each panel represents the Pearson correlation, and the blue border represents the distance correlation. Approximately 10% of complex correlated data information would be lost using the criterion that the Pearson correlation coefficient must be greater than 0.5

Szekely et al. [33] verified that the value of the distance correlation is always less than the absolute value of the Pearson correlation for bivariate normal data. Nevertheless, the difference between the two values is small. From the discussion in “Distance correlation better fits complex relationships” section, the identification capabilities of the linear and monotonous relationships between genes in the Pearson correlation and distance correlation were similar. Therefore, we could estimate that a complex relationship existed between two random variables if the distance correlation value was larger than the Pearson correlation. Here the complex relations refers to the non-bivariate normal data and non-linear and non-monotonic relations. Generally, the correlation value greater than 0.8 is described as strong correlation while the value less than 0.5 is described as weak correlation [34]. To measure the proportion of complex relationships in the microarray and RNA-seq datasets, we selected pairs of genes with distance correlation coefficients greater than 0.5 from the four datasets. Next, we analysed the distribution of Pearson correlation coefficients of the reserved pairs of genes. Of the total gene pairs, 9.98% had Pearson correlation coefficients less than 0.5 in the macrophage dataset (Fig. 5a). Moreover, the ratios in the liver dataset, (Fig. 5b), the cervical cancer dataset (Fig. 5c) and pancreatic cancer dataset (Fig. 5d) were 10.61%, 6.09% and 12.35%, respectively.

The cut-off threshold was used in the gene co-expression network [35, 36]. If 0.5 was used as the correlation coefficient cut-off threshold, approximately 10% of the complex correlated data information would be lost using the criterion that the Pearson correlation coefficient must be greater than 0.5. In WGCNA, soft-thresholding power was used, which amplifies disparity between strong and weak correlations. When the Pearson coefficient was used, these complex relationships achieved a smaller value, which would be further weakened by the soft threshold, resulting in a small weight of the two genes and inaccurate clustering results.

### Distance correlation shows evidence of SFT

Studies reported by Barabasi and Albert have suggested that the underlying topology of biological networks is approximately ’scale-free’ [37, 38]. It is important to identify hubs which dominate the SFT networks, such as genes, proteins, and metabolites, since they usually have great biological significance [39–42]. Therefore, we investigated the SFT of the two correlation coefficients for the four datasets.

**Fig. 6:**
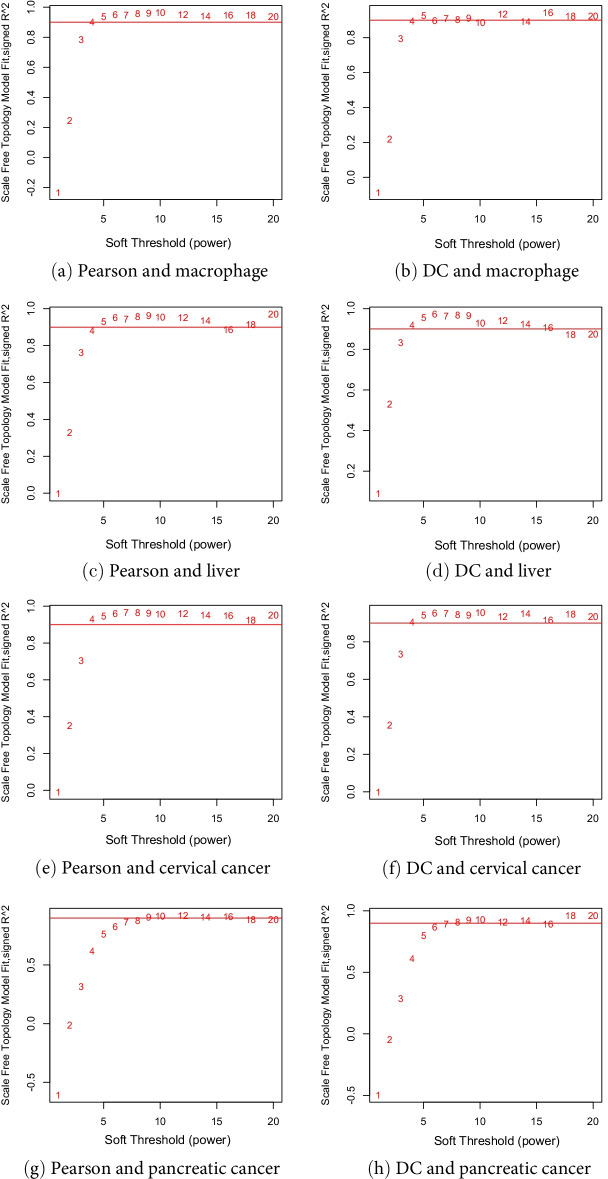
Distance correlation returns a scale-free network structure. Pearson correlation and distance correlation both indicate a degree of SFT; however, distance correlation shows slight advantages in the scale-free fit index compared with Pearson correlation for the liver and pancreatic cancer dataset

Soft-thresholding powers are key parameters in the construction of a gene co-expression network. In Fig. 6, we plotted the scale-free fit index (y-axis) of the Pearson-based WGCNA (see Fig. 6a, c, e, g) and DC-WGCNA (see Fig. 6b, d, f and h) corresponding to the soft-thresholding power (x-axis) for the four datasets. The closer the scale-free fit index is to 1, the better is the scale-free network. It was previously recommended to choose the soft-thresholding power when the scale-free fit index first reaches 0.9 [37], which is represented by the red horizontal line.

For macrophage and liver datasets, SFT was achieved when we raised the similar matrix to the 4 power for both Pearson and distance correlation. Moreover, for the liver dataset, the scale-free fit index of the Pearson correlation was less than 0.9, and the scale-free fit index of the distance correlation was greater than 0.9 when the recommended soft-thresholding power was 4. For the cervical cancer dataset, For both Pearson and distance correlation, after raising the correlation matrix to the power of 4, the SFT was achieved. The scale-free fit index of the Pearson correlation was greater than the distance correlation when the recommended soft-thresholding power was 4. Likewise, the pancreatic cancer dataset requires the power of 7 and 6. The scale-free fit index of the Pearson correlation was less than that of the distance correlation for the same soft-thresholding power.

Generally for the datasets, Pearson correlation and distance correlation could both achieve SFT with no significant difference. However, distance correlation showed slight advantages in the scale-free fit index compared with Pearson correlation for the liver and pancreatic cancer datasets when using the same soft-thresholding power.

### Cluster trees and modules

**Fig. 7:**
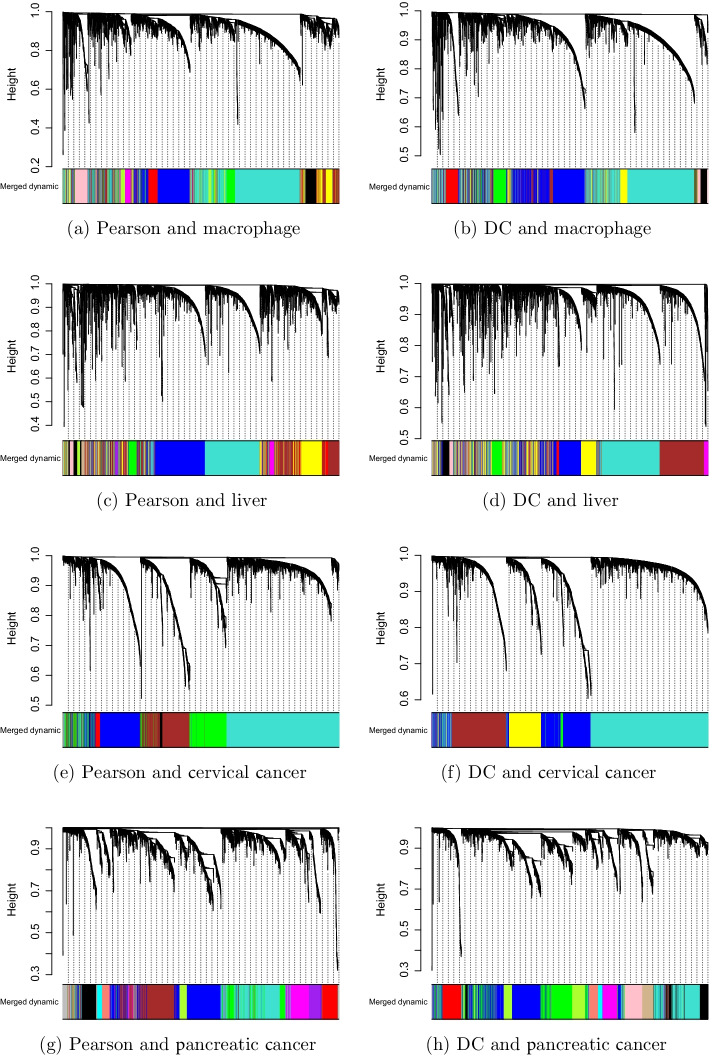
Cluster trees corresponding to Pearson-based WGCNA and DC-WGCNA. The cluster trees identified by DC-WGCNA and Pearson-based WGCNA are different, so the modules differ

To illustrate the impact of the different correlation measures on the gene co-expression network, we assigned the same parameter for Pearson-based WGCNA and DC-WGCNA with different correlation coefficients only. The cluster trees (dendrograms) of the two algorithms for the four datasets are shown in Fig. 7. The numbers of genes in each module are given in Additional file 1: Table S1.

In Fig. 7, modules are shown in different colours. For the macrophage dataset using Pearson-based WGCNA, 11 co-expression modules were constructed with 3,611 genes from 316 samples; however, this number was 9 using DC-WGCNA. For the liver dataset using Pearson-based WGCNA, 13 co-expression modules were constructed with 3,089 genes from 288 samples; however, this number was 10 using DC-WGCNA. For the cervical cancer dataset, using Pearson-based WGCNA, 7 co-expression modules were constructed with 3,808 genes from 308 samples; this number was 6 using DC-WGCNA. For the pancreatic cancer dataset, 12 co-expression modules were constructed with 3,029 genes from 183 samples both using Pearson-based WGCNA and DC-WGCNA. Thereinto, the grey module composed of genes that were not assigned to any gene co-expression module. The only obvious difference between these two algorithms was the correlation matrix when the input data and the other parameters were kept the same. As shown in Fig. 7, the number of modules identified by DC-WGCNA was usually less than that identified by Pearson-based WGCNA.

It can be intuitively seen that the cluster trees, module size, and cluster results obtained by the gene co-expression network analysis were completely different when the correlation coefficients were different and all other parameters identical. These findings indicate that the selection of correlation coefficients has a significant influence on the results of the gene co-expression network. Therefore, choosing an appropriate correlation coefficient is very important for the construction of a gene co-expression network.

### Gene enrichment comparison

Co-expressed genes are often involved in the same biological processes [43]. Therefore, the modules highly enriched for specific gene categories are more reasonable [18]. To compare the average enrichment score and stability of the algorithms, we assigned the appropriate values to the deepSplit and minClusterSize parameters in cutreeDynamic functions of the WGCNA package to make the module numbers obtained by WGCNA based on different correlation coefficients equal.

In the present analysis, we took the Top 3 enrichment scores in the Functional Annotation Clustering of DAVID [44–46]. The higher the enrichment score, the lower the P-value, thus the greater was the enrichment. The enrichment score of modules is an important index to appraise the rationality of modules. We will discuss the average enrichment scores of the modules derived from the gene co-expression network constructed with four different correlation coefficients to measure the enrichment degree of the co-expression network.

From Table 3, the modules from DC-WGCNA had a higher average DAVID enrichment score for the three datasets. The DAVID enrichment score of each module can is given in Additional file 2: Table S2. The higher the DAVID enrichment score, the more reasonable is the module. Modules from DC-WGCNA had the highest average DAVID enrichment scores among the three datasets, second only to Pearson correlation in the liver datasets. Modules from MIC almost had the lowest average DAVID enrichment scores, which was consistent with the above analysis of statistical power.

**Table 3:** Average DAVID enrichment score for each dataset

	Macrophage datasets	Liver datasets	Cervical cancer	Pancreatic cancer
Pearson	4.9685	6.1470	11.0860	7.9590
Spearman	4.3323	4.7704	11.5940	6.4339
MIC	4.1027	3.9617	8.6950	7.2693
Dcorr	5.2633	5.8904	14.2780	9.7644

For the liver dataset, the modules from WGCNA had a higher average DAVID enrichment score. We considered this phenomenon to be due to the following two reasons. First, we observed many outliers in the liver dataset, similar to the result shown in Fig. 4a. In some cases, such an ‘outlier’ might actually be biologically meaningful [13]. Second, the enrichment score was influenced not only by the correlation coefficient but also by the randomness of input data, parameter selection, clustering algorithm, module size and the number of genes in modules.

To test whether the identified modules obtained by DC-WGCNA are biologically meaningful, take the cervical cancer samples as an example, the highly enriched (Top3) biological process (BP) terms in GO for network modules were summarized. The turquoise module is significantly enriched in categories “epidermis development” (p = 3.4E−14) which is correlated with Epithelial-Mesenchymal Transition in cervical cancer [47], “angiogenesis” (p = 2.3E−7) which is important early in cervical pathogenesis [48], “keratinocyte differentiation” (p = 1.8E−6) which is dependent by Human Papillomaviruses (HPV) lifecycle, and the cervical cancers are driven by HPV of the high-risk variety [49]. Overall, the enrichment terms show the biologically meaningful of the modules obtained by DC-WGCNA.

To compare the biological functions of the modules between the Pearson-based WGCNA and DC-WGCNA. For the cervical cancer dataset, we selected the highly enriched BP terms ($p<0.01$) in GO for network modules obtained by the two methods. For the same colour, we calculated the numbers of overlapped BP terms between two modules obtained by the two methods and the proportion of the overlapped BP terms in the BP terms obtained by Pearson-based WGCNA. Thereinto, the proportion of the turquoise module is 71.62% which shows that the potential biological functions of the modules obtained by different clustering methods are similar sometimes. Simultaneously, the proportion of the brown module is 7.14% which shows that the potential biological functions of the modules obtained by different clustering methods are distinct obviously.

### Stability analysis

**Fig. 8:**
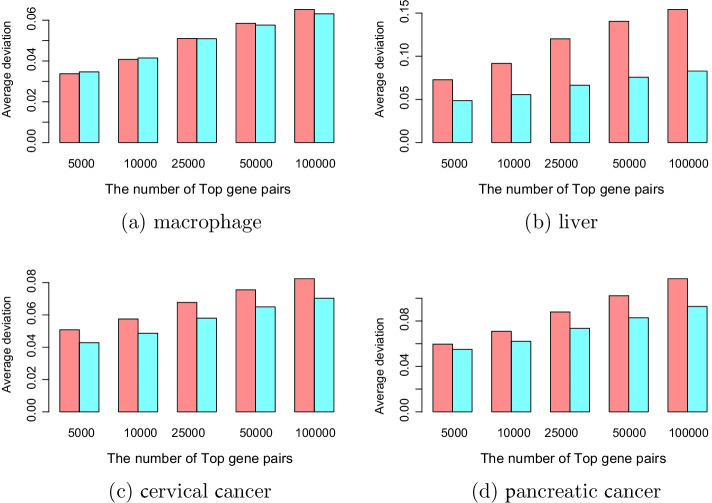
Stability based on highly correlated gene pairs. The red border in each panel represents the Pearson correlation. and the blue border represents the distance correlation. The lower the height of the border, the higher is the stability. In general, the stability of distance correlation was better than Pearson’s coefficient, and with an increasing number of top correlated gene pairs selected, the advantage of distance correlation becomes more obvious

In this section, we will compare the stability for highly correlated gene pairs and modules. First, we compare the stability based on highly correlated gene pairs. The microarray and RNA-seq datasets were divided into two halves with the same number of samples randomly, and calculated the correlation coefficients between different genes for each half independently. We calculated the Pearson coefficient of one half of the data and sorted these gene pairs according to the absolute Pearson value. The most correlated *n* ($n = 5000, 10{,}000, 25{,}000, 50{,}000, 100{,}000$) gene pairs were chosen. Then, we calculated the Pearson coefficient of these *n* gene pairs for the other half of the data. The stability of highly correlated gene pairs was demonstrated by comparing the mean absolute difference between the two halves of data. The smaller the mean absolute difference, the more stable it is. The same process was conducted for distance correlation. The results are shown in Fig. 8. For the macrophage dataset, Fig. 8a shows that the Pearson coefficient had good stability when the top number was small. With an increase in the top number, the stability of the distance correlation was stronger than the Pearson coefficient. For the liver data set, Fig. 8b shows that the stability of the distance correlation was significantly better than Pearson’s coefficient. Similarly, the advantage became more obvious as the top number increased. For the cervical cancer and pancreatic cancer datasets, the stability of the distance correlation was significantly better than the Pearson coefficient, which can be seen in Fig. 8c, d.

**Fig. 9:**
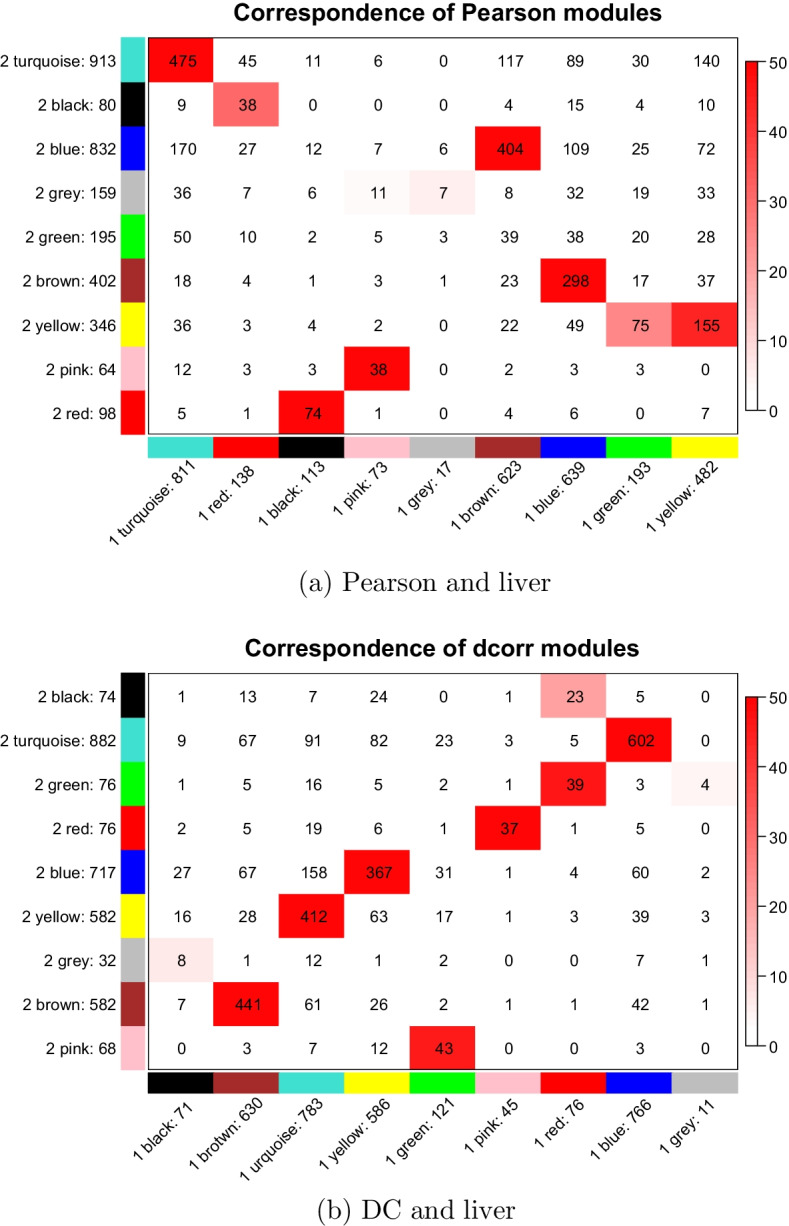
Module preservation between even partitioning of the liver dataset for (**a**) Pearson-based WGCNA and (**b**) DC-WGCNA, showing that DC-WGCNA has reasonable module preservation statistics. P-values are represented by the depth of the red colour; the darker the colour, the smaller is the p-value and the higher is the overlap

To determine the module stability, the liver data were randomly divided into two groups with the same number of samples, and each group of data was processed independently to obtain gene modules. To discuss the module preservation between the two parts of data, we examine the preservation significance (Fig. 9). The shade of the red colour here represents $-\log (p)$, where *p* is the Fisher’s exact test p-value for the overlap between the two modules. The shade of the red colour indicates the significant of the p-value; The darker the colour, the smaller the P-value, the more significant the overlap, and the more stable the module [37]. The numbers in Fig. 9 represent the gene counts in the corresponding module interaction. Since the gray module consists of genes that are not assigned to any module, it is not surprising that the grey module does not overlap with other modules.

We assigned appropriate parameters to make the module numbers obtained by WGCNA and DC-WGCNA equal to 9. The recommended soft-thresholding powers of WGCNA were 5 and 4, the number of the modules whose preservation significances larger than 50 was four (Fig. 9a). The red and green modules of part 1 of WGCNA showed relatively weak preservation. Moreover, the recommended soft-thresholding powers of DC-WGCNA were both 4. In Fig. 9b, five modules whose preservation significance is larger than 50 are shown. The black module of part 1 of DC-WGCNA showed relatively weak preservation, and there were fewer modules with weak overlap. For the liver dataset, DC-WGCNA was more stable than WGCNA in terms of preservation significance and suggested soft threshold. Other results from the other three datasets can be found in Additional file 3: Fig. S1. The results are similar to those obtained for the liver dataset,showing that DC-WGCNA was more stable than WGCNA. Since the division of the data was random, the gene modules were different. However, the stability did not change dramatically in either test.

## Discussion

In this paper, distance correlation was applied to WGCNA to construct a new gene co-expression network analysis method and the algorithm was verified to be valid using microarray and RNA-seq datasets.

We showed the complexity of gene relationships using gene expression microarray datasets and calculated the correlation coefficients of pairs of genes using different measures. The results showed that the distance correlation ability to detect various complex relationships between genes was strong. Moreover, Pearson correlation was too sensitive to outliers, while distance correlation was robust. Therefore, distance correlation is a good choice to measure the relationship between genes and discuss comparatively reasonable results.

To illustrate the efficiency of using distance correlation, we calculated the proportion of complex relationships in gene relationships when the distance correlation coefficient was greater than 0.5. Approximately 10% of relationships in the both microarray and RNA-seq datasets were complex.

To compare the performance of Pearson-based WGCNA and DC-WGCNA, we kept the other parameters constant and constructed a gene co-expression network using these two algorithms. DC-WGCNA showed slight advantages in the speed of achieving SFT and the scale-free fit index compared with Pearson correlation when the same soft-thresholding power was used. For further research, we calculated the DAVID enrichment score of the modules obtained using WGCNA based on four correlation measures. DC-WGCNA had a higher average DAVID enrichment score in most of the four datasets compared with the others. Moreover, considering the stability analysis, the modules from DC-WGCNA had better module preservation than those from Pearson-based WGCNA.

DC-WGCNA is significant, especially for gene datasets with complex relationships. However, the time complexity of distance correlation is $O(n^2)$ which is still a relatively expensive computation. The time complexity of distance correlation is greater than that of Pearson correlation of *O*(*n*). For example, the calculation of the distance correlation coefficients between approximately 3,000 genes in 300 samples by a PC (CPU: core i7) required approximately 9 hours. Moreover, according to the formula of distance correlation, an $n\times n$ distance matrix must be calculated. Thus, the memory required is large with a large number of samples. Fortunately, the number of samples analysed in this paper was small; therefore, the required memory was not vast. For the convenience of application, the R code used in this study has been provided in Additional file 4.

## Conclusions

Compared with the Pearson correlation coefficient, distance correlation is distribution free and better fits complex relationships. Its limitation is the large computation time and inability to determine positive and negative correlations. When outliers exist and the significant impact on the correlation coefficient value is undesirable, distance correlation is a better alternative choice. However, if these outliers are not errors and highlight their biological significance, we can choose the higher value between Pearson and distance correlations.

In this paper, we only discuss the applicability of distance correlation in gene co-expression network analysis. In fact, distance correlation is also suitable for measuring other complex relationships (such as proteins and metabolites), and we will attempt to apply distance correlation to other network analyses in our future work.

## Methods

### Distance correlation

Distance correlation was proposed in 2007 by Szekely, Rizzo, and Bakirov [17, 33]. For two random variables *X* and *Y*, the distance correlation coefficient is denoted as *R*(*X*, *Y*). And, $V_{n}(X,Y)$ denotes the empirical distance covariance which is a non-negative number defined by:

$$\begin{aligned} V_n^2(X,Y)=\frac{1}{n^2}\sum _{k,l=1}^nA_{kl} B_{kl} , \end{aligned}$$

Here, $A_{kl}$ and $B_{kl}$ are defined as $A_{kl}=a_{kl}-{\bar{a}}_{k.}-{\bar{a}}_{.l}+{\bar{a}}_{..}$ and $B_{kl}=b_{kl}-{\bar{b}}_{k.}-{\bar{b}}_{.l}+{\bar{b}}_{..}$, where $a_{kl}=\Vert X_k-X_l\Vert _p,\ b_{kl}=\Vert Y_k-Y_l\Vert _q$ with $k,l=1,\ldots , n$.

Likewise, for a random variable *X*, $V_n (X)$ is non-negative and defined as

$$\begin{aligned} V_n^2 (X)=V_n^2 (X,X)=\frac{1}{n^2} \sum _{k,l=1}^n A_{kl}^2. \end{aligned}$$

Finally, the empirical distance correlation $R_n (X,Y)$ is the square root of the following scheme

$$\begin{aligned} R_n^2 (X,Y)= \frac{V_n^2(X,Y)}{\sqrt{V_n^2 (X) V_n^2 (Y)}}. \end{aligned}$$

In this paper, we use the energy package in R to calculate the distance correlation (see the references in the manual for more details; https://CRAN.R-project.org/package=energy).

### Hypothesis testing and ROC curves

To evaluate the ability of the correlation coefficients for identifying the correlation between gene expression data, the concept of the receiver operating characteristic (ROC) curve was introduced based on the correlation coefficient hypothesis test. The test of dependence between *X* and *Y* is described as a hypothesis test as follows: $H_0$: *X* and *Y* are independent. $H_1$: *X* and *Y* are dependent. The definition of the ROC curves, the hypothesis testing, and realization methods used for the four correlation coefficients are referred to [28].

### Scale-free topology

Scale-free networks are complex networks which have a few highly connected nodes and most poorly connected nodes [50, 51]. The relationship between the topological properties of network nodes (genes and proteins) and functional essentiality in interaction networks is well-known [42, 52, 53].

SFT [4] has determined that the frequency distribution *p*(*k*) of connectivity number follows the power law: $p(k)\sim k^{-\gamma }$, where *k* is a non-negative real number. The weighted gene co-expression network conforms to SFT. Whether the network satisfies approximate SFT can be intuitively determined by drawing *log*(*p*(*k*)) versus *log*(*k*). The model fitting index $R^2$ is the squared of the correlation coefficient between *log*(*p*(*k*)) and *log*(*k*). There is a straight line between *log*(*p*(*k*)) and *log*(*k*) when $R^2$ of the model approaches 1.

The WGCNA R package [37] provides functions that help to choose the parameters (pickSoftThreshold), and the function scaleFreePlot help to evaluate whether the network exhibits SFT.

### WGCNA

WGCNA is a popular tool for identifying modules of highly correlated genes [4, 37, 54, 55]. The function of WGCNA is plentiful; only part has been used in this paper. We will introduce the process of module division of WGCNA. For other functions, we refer to the tutorials for the WGCNA package at https://labs.genetics.ucla.edu/horvath/CoexpressionNetwork/Rpackages/WGCNA/Tutorials/. The method used for network construction proceeded as follows. First, a similarity co-expression matrix was calculated with Pearson or another correlation coefficient *cor*(*i*, *j*) for all gene expression profiles. In this paper, we focused on the unsigned network in which the correlation coefficients are changed into absolute values. Next, an adjacency matrix is obtained from the similarity co-expression matrix by using the soft-thresholding power. The power is selected according to the criteria of approximately fitting the SFT network. Then, from the adjacency matrix, a topological overlap matrix is obtained. Based on the dissimilarity topological overlap matrix, a dendrogram was generated by using the hierarchical clustering method. Finally, different numbers of modules were obtained by dynamic tree cutting.

### DAVID enrichment analysis

DAVID [45, 46] is a database of bioinformatics resource which is available at http://david.abcc.ncifcrf.gov/. In this paper, the enrichment score of the DAVID Functional Annotation Clustering report is used. The report combines annotation terms from 14 public databases including KEGG pathways and Gene Ontology. The report makes the similar annotations class together and makes the biology clearer. The Group Enrichment Score is the geometric mean of the log p-values in the annotation term cluster, which is used to evaluate the enrichment significance of a gene module.

### Module stability

To evaluate the stability of the modules obtained by Pearson-based WGCNA and DC-WGCNA, one dataset was divided into two groups with the same number of samples randomly, and each group of data was processed independently to obtain gene modules to discuss the module preservation between the two parts. We calculated the overlaps of the two modules for each pair and used Fisher’s exact test to obtain a p-value for each of the pairwise overlaps. Module stability was processed by the R code (see [37]).

### Datasets

We evaluated the performance of this approach primarily on four different arrays (macrophage and liver) and RNA-seq (Cervical cancer and pancreatic cancer) datasets.

The macrophage and liver datasets were expression profiling by the array. We used 329 samples for macrophage dataset [22–24]. The liver dataset was gene expression in mice tissues consisting of 288 samples [25, 26]. Moreover, the data of both datasets were normalized using the RMA method. The macrophage and liver data can be downloaded from http://www.ncbi.nlm.nih.gov/geo/ under series GSE38705 and GSE16780, respectively.

The computation complexity of distance correlation is large $O(n^2)$. To overcome this computational difficulty and obtain the distance correlation coefficient, it is crucial to screen genes and limit the number of genes screened to a small number. From a biological point of view, a gene must be expressed to some extent before it can be translated into protein or considered biologically significant [56], and low-expression genes may be difficult to discern from noise [57]. Thus, we removed the genes with low expression by filtering. Simultaneously, we selected the genes whose expression varied significantly, since the genes without variation are uninformative for network analysis [18]. We calculated the coefficient of variation for each probe set. Then, the dataset will be reduced by selecting probes beyond the average intensity and the coefficient of variation (CV) is greater than 5%. In this way, we selected 3,611 genes for the macrophage dataset and 3,089 genes from the liver dataset for analysis.

Cervical cancer and pancreatic cancer datasets from Gene expression RNA-seq were performed using TCGA: https://www.cancer.gov/tcga. About the technique of obtaining cervical cancer and pancreatic cancer datasets, we refer to [58, 59]. The gene expression data of the datasets are transformed $log_2(x+1)$ and RSEM normalized count. Then, we calculated the average and CV of the gene expression profiles. And, the dataset was reduced by selecting probes beyond the average intensity and CV is greater than 10%. We selected 3808 genes in 308 samples for the cervical cancer dataset. For the pancreatic cancer dataset, there are 3029 genes in 183 samples.

## Supplementary information

**Additional file 1: Tabls S1.** The number of genes in each module.

**Additional file 2: Tabls S2.** The gene number and DAVID enrichment score in each module.

**Additional file 3: Fig. S1.** Module preservation between even partitionings of datasets.

**Additional file 4. Code S1.** The R code of the study.

## Data Availability

The macrophage and liver datasets can be download at http://www.ncbi.nlm.nih.gov/geo/ under accession numbers GSE38705 and GSE16780, respectively. Cervical cancer and pancreatic cancer datasets from Gene expression RNA-seq were performed using TCGA: https://www.cancer.gov/tcga. The R code associated with this study is published in one of the supplementary materials.

## Abbreviations

WGCNA: Weighted gene co-expression network analysis
DC-WGCNA: Distance correlation-based weighted gene co-expression network analysis
MIC: Maximal information coefficient
MICA: Maximal information component analysis
HMDP: Hybrid mouse diversity panel
RMA: Robust multi-array average
TCGA: The Cancer Genome Atlas
SFT: Scale-free topology
QQ: Quantile quantile
ROC: Receiver operating characteristic
LOF: Local outlier factor
EASE: Expression analysis systematic explorer
DAVID: Database for annotation, visualization and integrated discovery
GO: Gene Ontology
BP: Biological process
HPV: Human Papillomaviruses
KEGG: Kyoto encyclopedia of genes and genomes
OxPAPC: Oxidized 1-palmitoyl-2-arachidonoyl-sn-glycero-3-phosphorylcholine
TCGA: The cancer genome atlas
RSEM: RNA-seq by expectation-maximization
CV: Variation coefficient
CRAN: Comprehensive R archive network
