# Supplementary material for: Distance correlation application to gene co-expression network analysis
Source: BMC Bioinformatics. 2022 Feb 21;23:81. doi: 10.1186/s12859-022-04609-x (PMC8862277; doi:10.1186/s12859-022-04609-x)
Supplement: Supplementary file 1 — Additional file 1: Tabls S1. The number of genes in each module. [file 12859_2022_4609_MOESM1_ESM.pdf]

Table S1: The number of genes in each module

Macrophage and DC-WGCNA

black	blue	brown	green	grey	pink	red	turquoise	yellow
142	981	289	213	25	61	180	1437	283

Macrophage and Pearson-based WGCNA

black	blue	brown	green	greenyellow	grey	magenta	pink	red	turquoise	yellow
177	609	336	242	133	12	129	175	180	1356	262

Liver and DC-WGCNA

black	blue	brown	green	grey	magenta	pink	red	turquoise	yellow
90	624	611	118	77	42	64	96	832	535

Liver and Pearson-based WGCNA

black	blue	brown	green	greenyellow	grey	magenta	pink	purple	red	tan	turquoise	yellow
91	674	645	108	46	36	57	60	48	93	38	754	439

Cervical cancer and DC-WGCNA

blue	brown	green	grey	turquoise	yellow
807	767	54	10	1696	474

Cervical cancer and Pearson-based WGCNA

black	blue	brown	green	grey	red	turquoise
64	677	670	686	19	74	1618

Pancreatic cancer and DC WGCNA

black	blue	cyan	green	greenyellow	grey	magenta	pink	red	salmon	tan	turquoise
211	682	64	497	319	10	173	201	214	113	125	420

Pancreatic cancer and Pearson-based WGCNA

black	blue	brown	cyan	green	greenyell	grey	magenta	purple	red	salmon	turquoise
179	614	498	68	244	124	102	290	141	182	101	486
