# Supplementary material for: Distance correlation application to gene co-expression network analysis
Source: BMC Bioinformatics. 2022 Feb 21;23:81. doi: 10.1186/s12859-022-04609-x (PMC8862277; doi:10.1186/s12859-022-04609-x)
Supplement: Supplementary file 2 — Additional file 2: Tabls S2. The gene number and DAVID enrichment score in each module. [file 12859_2022_4609_MOESM2_ESM.pdf]

Table S2: The gene number and DAVID enrichment score in each module

Macrophage and Pearson-based WGCNA

Module colour	black	blue	brown	green	magenta	pink	red	turquoise	yellow	grey
number of gene	175	652	365	287	135	130	183	1350	322	12
Cluster 1 score	6.03	5.63	3.97	7.37	12.41	5.58	2.21	10.19	12.49	0.21
Cluster 2 score	4.54	3.86	2.33	4.72	5.62	1.43	2.16	6.46	6.64	
Cluster 3 score	2.98	3.71	1.82	4.68	4.26	0.98	1.65	5.08	5.35	

Macrophage and Spearman-based WGCNA

Module colour	black	blue	brown	green	greenyellow	magenta	midnightblue	purple	salmon	turquoise
number of gene	557	735	438	176	64	93	30	79	102	1337
Cluster 1 score	7.92	5.46	2.79	4.93	1.94	4.59	2.85	2.32	12.89	13.38
Cluster 2 score	6.82	4.8	2.72	2.98	1.74	3.4	2.81	1.74	4.68	8.74
Cluster 3 score	5.63	4.49	2.47	1.97	0.95	2.54	2.43	1.41	3.73	4.85

Macrophage and MIC-based WGCNA

Module colour	blue	brown	greenyellow	magenta	pink	purple	red	salmon	tan	turquoise
number of gene	989	127	12	15	16	13	225	12	12	2190
Cluster 1 score	6.28	5.37	4.19		2.99	1.4	12.09	2.64	3.15	11.96
Cluster 2 score	4.61	3.95	2.43		2.85	1.15	8.1	1.99	0.5	8.77
Cluster 3 score	4.08	2.2			0.89	0.5	7.59	0.4	0.24	6.35

Macrophage and DC-WGCNA

Module colour	black	blue	brown	green	magenta	pink	red	turquoise	yellow	grey
number of gene	141	936	281	199	61	93	177	1420	279	24
Cluster 1 score	5.92	6.03	2.54	11.02	10.68	3.35	6.85	13.9	5.39	0.85
Cluster 2 score	2.87	4.36	2.36	4.45	9.47	2.17	5.31	6.63	4.68	0.57
Cluster 3 score	1.48	4.21	2.12	4.14	7.67	1.95	2.96	6.53	3.07	

Liver and Pearson-based WGCNA

Module colour	black	blue	brown	green	magenta	pink	red	turquoise	yellow	grey
number of gene	86	712	697	115	41	68	97	777	449	47
Cluster 1 score	12.62	8.6	8.76	5.59	4.1	6.88	15.56	9.53	8.06	1.6
Cluster 2 score	4.51	4.63	8.72	3.27	3.37	4.02	9.14	8.43	4.64	0.88
Cluster 3 score	3.97	4.56	5.05	1.83	1.96	3.48	6.62	4.76	3.31	0.36

Liver and Spearman-based WGCNA

Module colour	black	blue	brown	green	magenta	pink	red	turquoise	yellow	grey
number of gene	68	684	607	116	33	44	107	890	513	27
Cluster 1 score	4.2	7.81	8.5	3.98	2.72	0.6	16.06	8.75	6.87	1.65
Cluster 2 score	3.29	5.77	6.22	2.05	0.94	1.67	5.08	7.19	5.28	1.23
Cluster 3 score	2.82	3.88	4.59	1.62		0.61	3.84	5.87	3.82	0.68

Liver and MIC-based WGCNA

Module colour	black	blue	brown	green	magenta	pink	purple	red	turquoise	yellow
number of gene	30	916	579	40	24	26	23	30	989	432
Cluster 1 score	5.07	8.86	7.43	3.64	0.59	3.03	1.92	3.43	6.92	11.77
Cluster 2 score	4.1	5.79	5.44	3.18	0.26	2.93	0.72	2.91	6.76	6.97
Cluster 3 score	2.62	3.26	3.93	2.04	0.25	2.53	0.27	2.84	5.05	4.34

Liver and DC-WGCNA

Module colour	black	blue	brown	green	magenta	pink	red	turquoise	yellow	grey
number of gene	90	624	611	118	42	64	96	832	535	77
Cluster 1 score	14.93	8.98	9.33	3.22	2.37	5.72	15.53	10.25	8.31	1.61
Cluster 2 score	9.6	3.76	6.95	2.45	0.66	3.85	10.69	7.44	3.49	1.43
Cluster 3 score	3.38	3.58	4.22	1.81	0.59	2.76	7.38	4.86	2.93	1.41

Cervical cancer and Pearson-based WGCNA

Module colour	blue	brown	green	turquoise	yellow	grey
number of gene	795	697	82	1642	570	22
Cluster 1 score	13.6	10.93	10	10.77	35.6	2.84
Cluster 2 score	12.91	8.26	6.85	5.22	21.29	2
Cluster 3 score	8.14	2.53	3.68	4.5	12.01	0.21

Cervical cancer and Spearman-based WGCNA

Module colour	blue	brown	green	turquoise	yellow	grey
number of gene	782	645	49	1815	516	1
Cluster 1 score	17.89	21.27	18.39	9.23	36.18	
Cluster 2 score	8.47	2.61	4.71	4.5	21.72	
Cluster 3 score	7.97	2.44	3.56	3.78	11.19	

Cervical cancer and MIC-based WGCNA

Module colour	black	blue	brown	red	turquoise	yellow
number of gene	22	917	446	29	2034	360
Cluster 1 score	2.34	5.38	41.64	17.73	9.75	3.33
Cluster 2 score	1.77	4.9	22.23	3.93	8.26	3.29
Cluster 3 score	1.45	4.68	12.02	3.32	7.84	2.65

Cervical cancer and DC-WGCNA

Module colour	blue	brown	green	turquoise	yellow	grey
number of gene	807	767	54	1696	474	10
Cluster 1 score	17.51	21.73	23.17	10.67	41.56	0.54
Cluster 2 score	7.88	6.07	4.61	5.79	37.99	
Cluster 3 score	6.9	2.42	4.6	4.39	18.88	

Pancreatic cancer and Pearson-based WGCNA

Module colour	black	blue	brown	green	greenyellow	grey60	lightgreen	lightyellow	midnightblue	purple	tan	turquoise	yellow	grey
number of gene	424	930	1127	1686	420	137	87	82	155	364	243	947	537	708
Cluster 1 score	8.94	7.17	5.6	31.47	5.88	2.85	6.41	6.41	8.75	7.37	6.41	7.44	37.17	2.56
Cluster 2 score	2.38	5.88	4.47	22.14	2.99	2.4	3.18	3.18	7.11	5.46	3.18	4.24	30.45	2.37
Cluster 3 score	1.84	5.53	4.42	17.85	2.88	1.96	2.78	2.78	4.82	4.13	2.78	2.84	16.86	2.34

Pancreatic cancer and Spearman-based WGCNA

Module colour	black	cyan	darkgreen	darkred	green	greenyell	grey60	lightcyan	magenta	purple	red	royalblue	salmon	yellow
number of gene	593	101	23	24	617	496	43	100	334	146	184	29	110	229
Cluster 1 score	16.47	40.2	11.58	0.59	9.47	35.25	1.04	2.76	6.07	8.69	2.2	0.86	1.98	13.03
Cluster 2 score	10.27	14.01	3.6	0.37	8.5	8.19	0.14	2.11	3.35	4.51	1.46	0.23	1.2	10.4
Cluster 3 score	6.75	9.17	2.45	0.27	3.37	7.44	0.11	1.46	3.02	2.58	1.15		1.12	6.37

Pancreatic cancer and MIC-based WGCNA

Module colour	black	blue	brown	green	greenyellow	grey60	lightcyan	lightgreen	pink	purple	red	salmon	turquoise	yellow
number of gene	150	380	292	172	89	25	117	24	101	92	157	96	1134	200
Cluster 1 score	4.95	6.65	12.61	6.67	1.66	1.06	29.19	13.37	1.95	42.07	4.37	6.3	20.37	13.05
Cluster 2 score	4.37	5.94	4.78	3.25	1.51	0.03	22.55	3.51	1.62	14.59	2.8	1.9	15.35	6.84
Cluster 3 score	3.94	3.54	3.74	3.19	0.83	0.02	8.14	0.54	1.57	9.77	2.58	1.49	8.88	3.77

Pancreatic cancer and DC-WGCNA

Module colour	black	blue	brown	cyan	greenyellow	grey60	magenta	midnightblue	pink	royalblue	salmon	tan	turquoise	grey
number of gene	1304	745	1166	272	809	195	910	242	376	94	307	318	760	349
Cluster 1 score	4.47	11.83	7.66	3.41	29.08	3.92	26.21	58.24	8.55	31.71	4.82	11.85	9.29	2.29
Cluster 2 score	3.08	5.44	4.95	3.1	16.02	3.38	11.32	20.04	1.52	14.41	3.06	5.84	3.69	1.42
Cluster 3 score	2.53	3.85	4.75	3.02	8.02	3.22	9.12	16.37	1.5	11.45	3	3.69	3.4	1.31
