# Supplementary material for: Distance correlation application to gene co-expression network analysis
Source: BMC Bioinformatics. 2022 Feb 21;23:81. doi: 10.1186/s12859-022-04609-x (PMC8862277; doi:10.1186/s12859-022-04609-x)
Supplement: Supplementary file 3 — Additional file 3: Fig. S1. Module preservation between even partitionings of datasets. [file 12859_2022_4609_MOESM3_ESM.pdf]

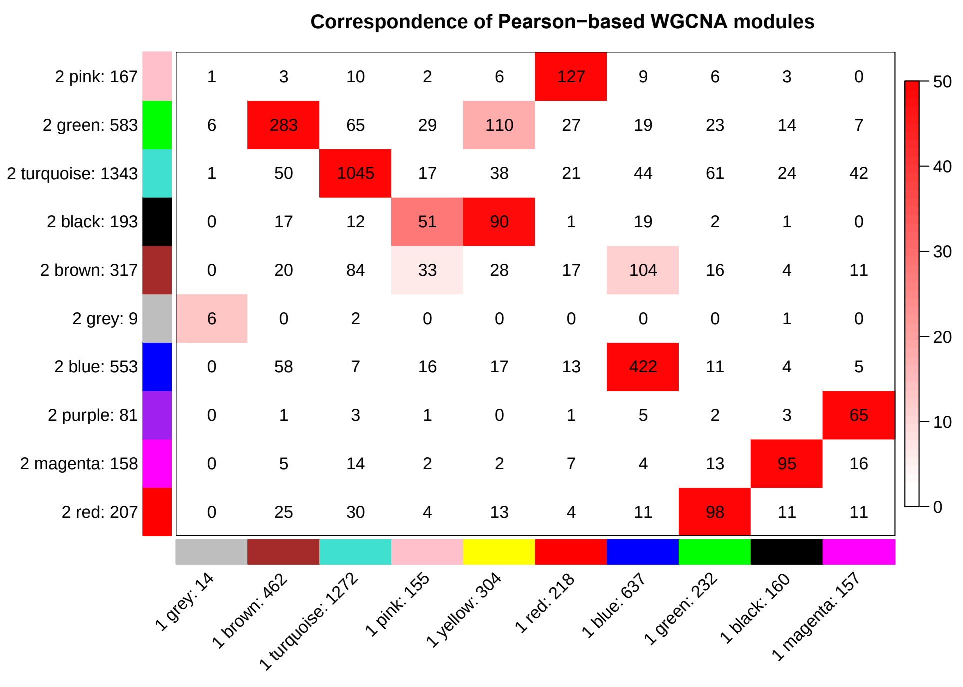

(a) Pearson and macropahge

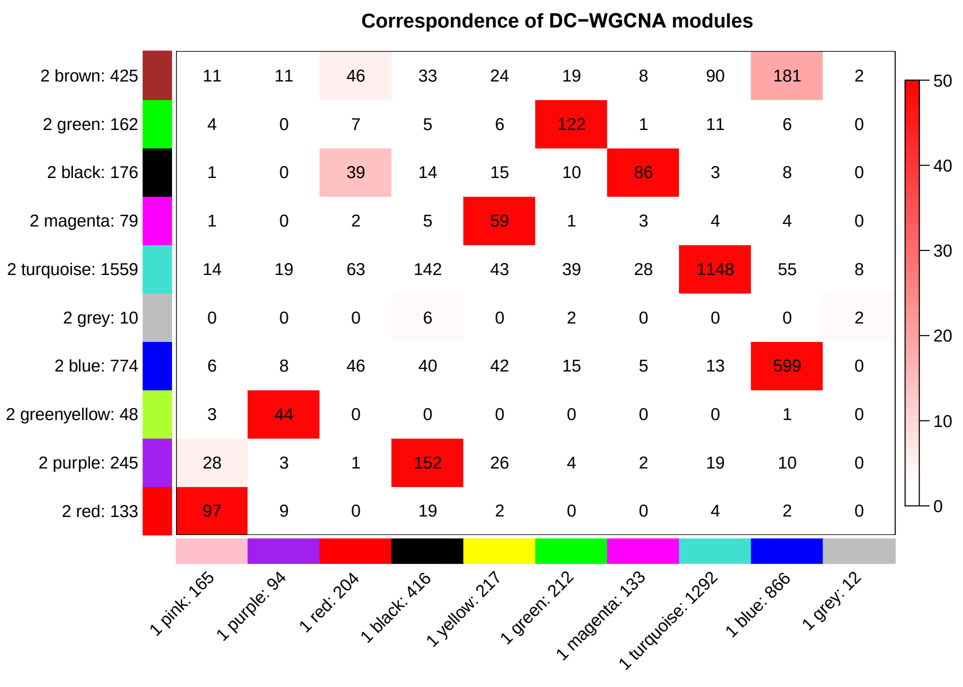

(b) DC and macropahge

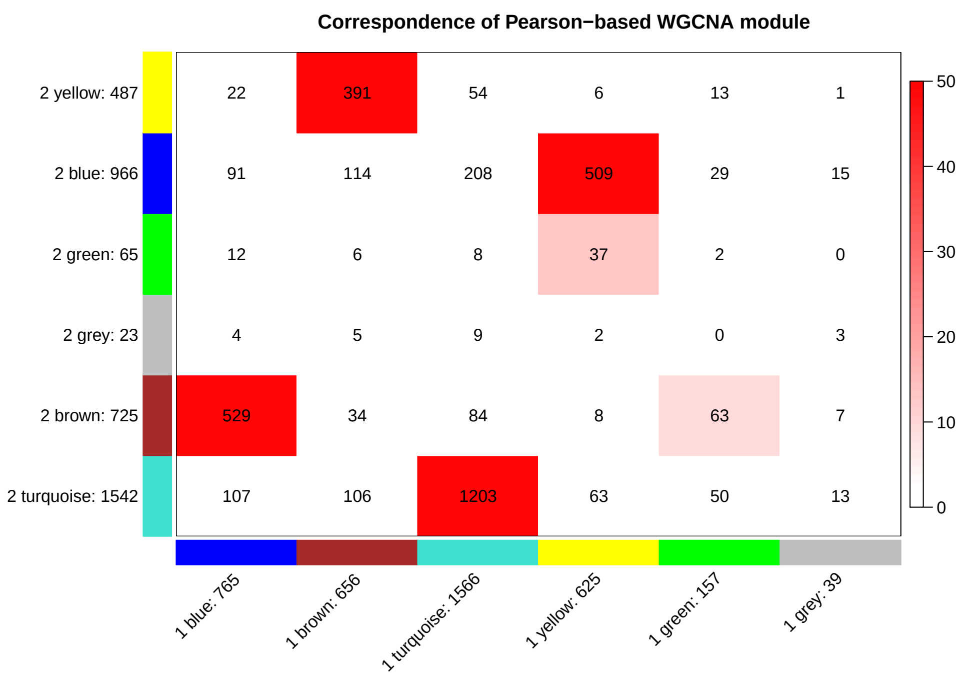

(c) Pearson and cervical cancer

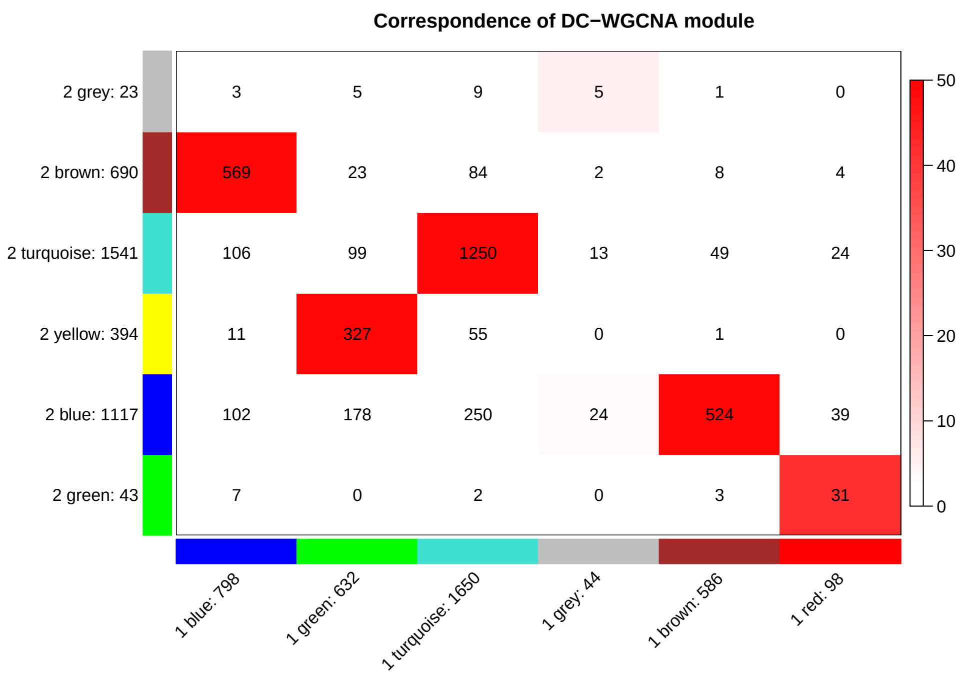

(d) DC and cervical cancer

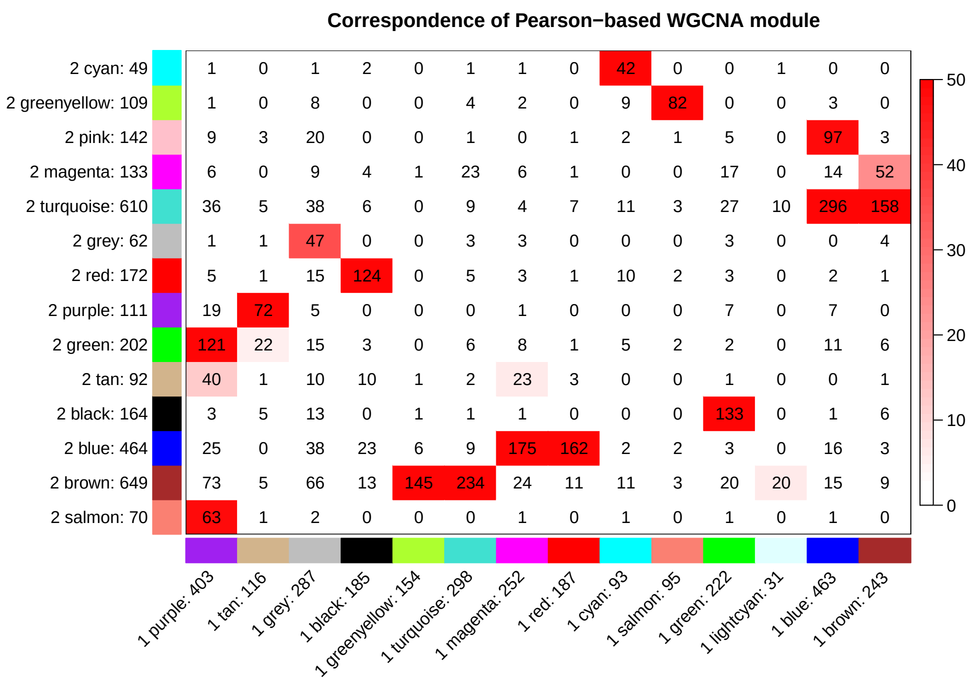

(e) Pearson and cervical cancer

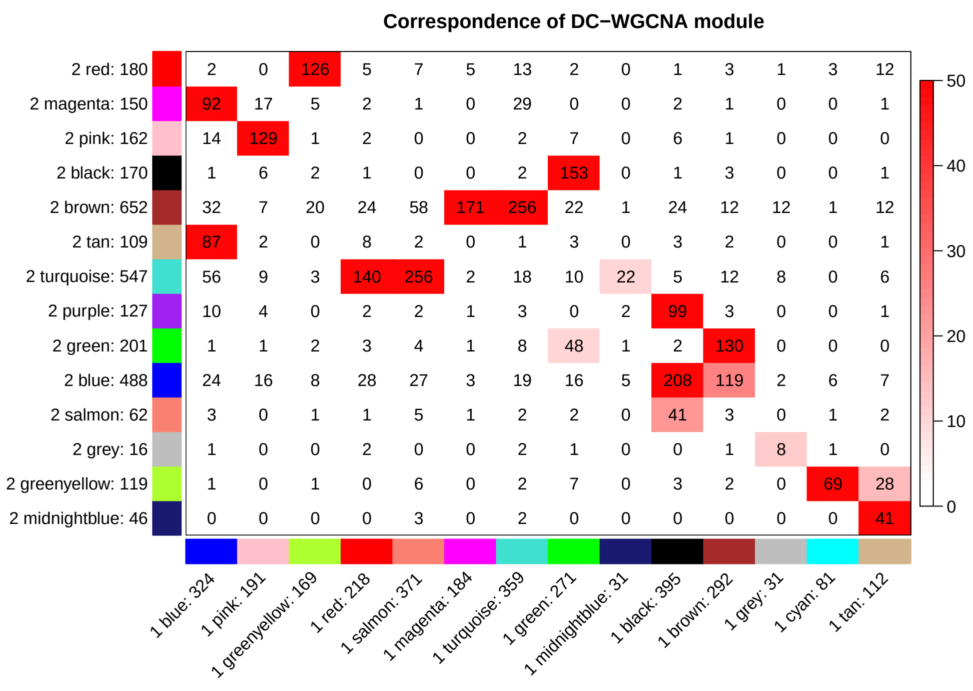

(f) DC and cervical cancer

Figure s1: The module preservation between each partition of datasets. The numbers of modules with preservation significances greater than 50 are 7 vs 8 in macrophage, 4 vs 4 in cervical cancer, 12 vs 14 in pancreatic cancer for Pearson-based WGCNA compared with DC-WGCNA.
